# Assistive Technology Innovations in Neurological Conditions

**DOI:** 10.1155/2021/6846120

**Published:** 2021-02-20

**Authors:** Carlos Bandeira de Mello Monteiro, Helen Dawes, Nancy Mayo, Johnny Collett, Fernando Henrique Magalhães

**Affiliations:** ^1^Grupo de Pesquisa em Aplicações Tecnológicas em Reabilitação School of Arts, Sciences and Humanities-EACH, University of São Paulo, São Paulo, SP, Brazil; ^2^Centre for Movement, Occupational and Rehabilitation Sciences, Oxford Brookes University, Oxford, UK; ^3^NIHR Oxford Health Biomedical Research Centre, Canada; ^4^McGill University, Québec, Canada

Advances in assistive technologies, aimed at maintaining or improving individuals' function and independence, thereby promoting their well-being [1, 2], have led to improvements in the autonomy and quality of life of people with neurological disabilities [3]. Nevertheless, the process of translating assistive devices from the laboratory bench to the manufacture of accessible products that meet the needs of people with neurological impairments is undoubtedly challenging as there is an ongoing need for reinvestment in science and technology and in the validation process [4].

This special issue provides recent developments in and summarizes studies of the development, testing, and application of assistive technology innovations in neurological conditions. High-quality research articles and systematic reviews strive to inform and investigate how assistive technologies can enable individuals with a variety of neurological conditions in many different aspects of their lives. Topics included in the issue address the use of assistive technology for improving health, psychological, and social status, as well as motor/cognitive learning and performance.

More specifically, the systematic review by B. Thordardottir et al. synthesized studies on facilitators and barriers related to the acceptance and use of innovative assistive technologies among people with cognitive impairments and their caregivers. E. Bartkiene et al. used sensory traits and face reading technology to analyse several factors (such as social status, age, gender, education, knowledge about healthy eating, and attitude to food) affecting consumer food choices. C. L. Kwan et al. investigated a wearable assistive device based on patterns of physiological signal changes and found it was able to successfully identify moments of significance experienced by individuals with dementia and their caregivers. The systematic review by A.V. L. de Araujo et al. evaluated the effectiveness of virtual reality-based rehabilitation interventions after spinal cord injury. C. Luque-Moreno et al. provided evidence for the reduction of spasticity and improvement of gait function in poststroke individuals who had traditional rehabilitation augmented with virtual reality reinforcing feedback. Finally, the results of A. H. Moliterno et al. suggested that applying computer game-based randomized training (compared to constant practice) might have superior effects on the motor performance of individual poststroke.

This special issue illustrates the wide variety of technologies that have been applied to assist people with various neurological conditions. Incorporating such a range of topics is certainly one of the strengths of this issue, which offers a broad overview on many different innovations in assistive technologies designed to improve physical functioning and promote well-being in neurologically impaired individuals. The included studies have all succeeded in addressing the potential of the technological aspects of different tools/devices, the methods used in their evaluation, and have provided results that advance the current knowledge of the mechanisms behind the associated conditions, which is a crucial role in developing understanding of the effects of the proposed interventions.

In conclusion, this special issue outlines recent developments in knowledge on many different aspects of assistive technologies and their application for individuals with neurological impairments. Future research must continue to focus on the development, evaluation, and application of assistive technology innovations in neurological conditions, to provide enhanced knowledge about the aspects involved in the associated conditions and hence pave the way to build successful strategies to improve an individual's function and independence.

*Carlos Bandeira de Mello Monteiro* *Helen Dawes* *Nancy Mayo* *Johnny Collett* *Fernando Henrique Magalhães*
